# Ginsenoside Rh2 administration produces crucial antidepressant‐like effects in a CUMS‐induced mice model of depression

**DOI:** 10.1002/brb3.2705

**Published:** 2022-07-18

**Authors:** Lin‐Sheng Shi, Chun‐Hui Ji, Yue Liu, Jiang‐Hong Gu, Wen‐Qian Tang, Wei Zhang, Wei Guan

**Affiliations:** ^1^ Department of Cardiology Affiliated Hospital 2 of Nantong University Nantong China; ^2^ Department of Pharmacology Pharmacy College, Nantong University Nantong China; ^3^ Provincial key laboratory of Inflammation and Molecular Drug Target Nantong China; ^4^ School of Medicine Nantong University Nantong China

**Keywords:** brain‐derived neurotrophic factor, chronic unpredictable mild stress, depression, ginsenoside Rh2

## Abstract

**Introduction:**

The most striking feature of depression is sadness and a loss of interest in activities, which represents a major cause of disability globally. Therefore, it is necessary to identify novel antidepressants for clinical practice. Ginsenoside Rh2 (Rh2) is one of the major bioactive ginsenosides that can be extracted from Panax ginseng and has been demonstrated to improve both memory and learning. The purpose of this study was to provide broad insight into the mechanisms underlying depression and gain greater insights into antidepressant therapy.

**Methods:**

In this study, we first established an effective and feasible depression animal model of chronic unpredictable mild stress (CUMS) and behavioral testing was evaluated by the forced swim test (FST), the tail suspension test (TST) and the sucrose preference test. Following pretreatment with Rh2 (10 and 20 mg/kg), the immobility time of mice was reduced without affecting locomotor activity in both the FST and TST. Western blotting and immunofluorescence were used to investigate the activation of the hippocampal BDNF signaling pathway and hippocampal neurogenesis.

**Results:**

Different concentrations of Rh2 significantly reduced depressive‐like symptoms in CUMS‐induced mice and downregulated the effects of the BDNF signaling cascade and neurogenesis in the hippocampus. Furthermore, the administration of K252a completely prevented the antidepressant‐like activity of Rh2 in mice.

**Conclusion:**

The results indicated that Rh2 possesses the antidepression action via the positive regulation of the BDNF‐TrkB pathway.

## Graphical Abstract

Graphical Abstract.

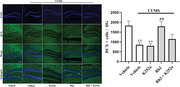

## 1 INTRODUCTION

Depression is a common clinical mood disorder that affects more than 350 million people at a high recurrence rate around the world, which results in heavy public health and a serious social burden (Ledford, 2014). However, most of the classic antidepressants used in the clinic at present are designed to improve the levels of monoamine neurotransmitters. These drugs can have some undesirable side effects, such as substantial side effects, slow onset, and high recurrence (Schechter et al., 2005). Thus, it is imperative and necessary to elucidate the pathogenesis of depression and develop novel drugs that are efficient and safe (Shafiee et al., 2018).

BDNF, a member of the neurotrophin family, plays a critical role in neurogenesis, neuronal survival, and brain homeostasis (Begni et al., 2017). BDNF and the mechanisms underlying its actions are currently being used to treat these diseases, such as antidepressants and antipsychotics (Autry & Monteggia, 2012). BDNF binds TrkB, a member of the family of Trk receptors, to regulate many different cellular processes involved in the development of brain function (Colucci‐D'Amato et al., 2020). For example, *Asparagus cochinchinensis* extract has been confirmed to exert antidepressant effects in an animal model of menopausal depression by activating the hippocampal BDNF‐TrkB pathway (Kim et al., 2020). The activation of AMPK produces a range of anti‐depressant effects that can mediate hippocampal neurogenesis via the BDNF/TrkB/CREB signaling pathways in neurons (Odaira et al., 2019). Recent animal studies have proven that chronic unpredictable mild stress can significantly reduce the expression of BDNF in the pathogenesis of depression (Duman et al., 2021; Gudasheva et al., 2019; Peng et al., 2018).

Evidence indicates that natural products can be an alternative and cost‐efficient complementary medicine for the treatment of depression, largely due to their multitarget efficacy and low toxicity (Chong et al., 2019). Ginseng is a traditional neuroprotective herbal medicine. The major active ingredients of ginseng are ginsenosides, which have been confirmed to improve cognitive and memory abilities in animal models (Kezhu et al., 2017; F. Li et al., 2016; Zhu et al., 2015). Ginsenoside Rh2 (Rh2) is one of the major bioactive ginsenosides from Panax ginseng. Rh2 has been demonstrated to possess antimetastasis, antiproliferation, and anti‐invasion effects and can promote differentiation (X. Li et al., 2020). In 2018, Lu et al. (2018) found that ginsenosides Rh2 may protect against the spatial and nonspatial memory deficits induced by SD mice. Later, Lv et al. (2021) demonstrated that Rh2 exhibited neuroprotective effects in a model of Scop‐induced memory dysfunction in mice. Thus far, there have been no investigations relating to the possibility of Rh2 as a potential antidepressant. Therefore, we investigated the mechanisms by which Rh2 can exert antidepressant actions in CUMS‐induced mice.

## 2 MATERIALS AND METHODS

### 2.1 Animals

Seven‐week‐old of adult male mice (20–22 g) were purchased from the Experimental Animal Centre of Medical College at Nantong University. Before the start of the study, the animals were divided into five groups per cage and kept under a 12‐h dark/light cycle, in a room temperature of 24 ± 1°C. Food and water were provided ad libitum for 1 week (Jiang et al., 2019). Prior to the experiments, the mice were acclimatized for 7 days. All animal protocols and procedures were approved by the Institutional Animal Ethical Committee of Nantong University (approval No., 20171220‐005) and conducted according to the NIH Guidelines.

### 2.2 Materials

The ginsenoside Rh2 (Rh2) was obtained from Puda Biological Technology Co. Ltd. (Suzhou, China). Fluoxetine was purchased from Sigma–Aldrich (St. Louis, MO, USA). K252a was obtained from Alomone Laboratories (Jerusalem, Israel). Rh2, fluoxetine and K252a were dissolved in 1% dimethyl sulfoxide in normal saline (vehicle) and intraperitoneally (i.p.) injected with different doses. The dosages of Rh2 (10 and 20 mg/kg), fluoxetine (20 mg/kg), and K252a (25 μg/kg) were chosen based on previous reports (Mu et al., 2019; J. J. Zhang, Gao, et al., 2019).

### 2.3 Chronic unpredictable mild stress

The chronic unpredictable mild stress (CUMS) procedure was performed as described with a slight modification (Mavrakis et al., 2010; J. Xu et al., 2015). Mice were subjected to a random sequence of unpredictable and mild stressors for 42 days. Different stressors are listed as follows: water or food deprivation (23 h), overnight illumination, restraint (2 h), cage shaking (15 min), tail pinching (2 min), remaining in the cage at a 45° tilt (17 h), and remaining in a soiled cage (5 h). After 4 weeks, the CUMS model was prepared, while administration of Rh2, fluoxetine, and K252a was performed during the last 2 weeks. The depression‐like behavior of the mice was detected using the forced swim test (FST), tail suspension test (TST), and sucrose preference test (SPT).

### 2.4 Forced swim test

FST was performed according to Jiang et al. with some modifications (Jiang, Huang, Chen, et al., 2015; Jiang, Huang, Zhu, et al., 2015; Ren et al., 2017; D. Xu et al., 2018). The equipment for the FST consisted of a transparent glass cylinder (45 cm height × 30 cm diameter) containing 22 cm of water (room temperature), and mice were floated in water for a total of 6 min. The total immobility time was recorded for the last 4 min by the automatic analyzer system. The water in the cylinder was replaced at the end of each trial.

### 2.5 Tail suspension test

The TST is another widely used pharmacological in vivo model for the evaluation of depression‐like behavior in mice. During the experiment, each mouse was individually suspended on a metal rod by tail 50 cm above the floor for 6 min. During the last 4 min, the immobility time of the mice was automatically recorded with behavioral software (Leng et al., 2018; Wu et al., 2018).

### 2.6 Open field test

An open field test (OFT) was conducted to eliminate the influence of mice locomotor activity and evaluated by the time spent in predefined central or peripheral areas (P. Xu et al., 2017). Mice were gently placed in the center of an open field (a wooden box, 100 × 100 × 40 cm) with its floor divided into 25 squares for 5 min. The total distance of the animals was recorded by the camera system for 5 min under dim light conditions. The apparatus ground was cleaned for each trial (Jiang, Huang, Zhu, et al., 2015; L. Y. Lin et al., 2016; D. Xu et al., 2018).

### 2.7 Sucrose preference test

Before the test, the mice were adapted to the environment with 1% (w/v) sucrose solution and tap water for 24 h (Stepanichev et al., 2016; Zhao et al., 2019). Afterward, the animals were not given food and water for 18 h, and then given preweighed bottles containing 1% sucrose solution and tap water for 6 h testing (two‐bottle test, 2‐h periods) (Gao et al., 2020; J. L. Wang, Wang, et al., 2020). Preference value is given as (%) = sucrose intake (g)/(sucrose intake (g) + water intake (g)) × 100%.

### 2.8 Western blot analysis

Total proteins from the hippocampus were homogenized on ice in 100 μl lysis buffer (Thermo Fisher Scientific, Waltham, MA, USA) plus protease inhibitors. The extract was centrifuged, and the supernatant was collected. Protein concentrations were measured by the BCA Protein Assay Kit (Beyotime Biotechnology, China). Equivalent amounts of protein were loaded in sodium dodecyl sulfate‐polyacrylamide gel electrophoresis (SDS‐PAGE) gels. Proteins were transferred to polyvinylidene difluoride membranes (Millipore, Billerica, MA, USA). The membranes were blocked with TBST containing 5% skim milk for 2 h at room temperature. Then, the membranes were treated overnight at 4°C with the different antibodies: β‐actin (1:2000; Cell Signaling), rabbit anti‐BDNF (1:500; Abcam), rabbit anti‐ERK1/2 (1:1000; Cell Signaling), rabbit anti‐pERK1/2 (1:500; Cell Signaling), rabbit anti‐AKT (1:1000; Abcam), rabbit anti‐pAKT (1:500; Abcam), rabbit anti‐CREB (1:1000; Cell Signaling), and rabbit anti‐pCREB (1:500; Cell signaling). Next, the membranes were incubated with the respective secondary antibodies for 2 h at room temperature. Then, the membrane was detected using enhanced chemiluminescence reagent (Guan et al., 2017, 2021).

### 2.9 Immunofluorescence

As we have frequently described (Jiang, Huang, Chen, et al., 2015; Jiang, Huang, Zhu, et al., 2015; Jiang et al., 2017), the mice from each group were anesthetized with 0.5% sodium pentobarbital (Macklin, Shanghai), pericardial perfused with 0.9% saline, and then fixed in 800 ml of 4% paraformaldehyde (PFA) overnight. The brain was removed and placed in 4% PFA for postfixation (24 h, 4°C). The brain was dehydrated in 30% sucrose (48 h, 4°C), and opti‐mum cutting temperature compound (OCT) was then embedded. Next, the 25‐μm‐thick sections were cut by using a Leica freezing microtome and immediately adhered to the slide. First, the slides were washed in phosphate‐buffered saline with 0.5% Triton X‐100 (Beyotime, China) for 5–10 min. Second, the slices were incubated in a blocking solution containing 3% bovine serum albumin (BSA) for 30 min at room temperature, and the following primary antibodies diluted in 3% BSA: DCX (1:100; Cell Signaling) were incubated overnight. Third, the FITC‐labeled secondary antibody (1:50; Thermo Fisher, USA) was added for 2 h at 37°C. Finally, the nuclei were stained with DAPI (Sun et al., 2020).

### 2.10 Statistical analysis

The statistical analyses and bar graphs were generated with GraphPad Prism 6.0. The differences among treatment groups were analyzed using SPSS software including one‐way or two‐way analysis of variance (ANOVA) as appropriate, by post hoc Bonferroni's test for multiple comparisons. The results were considered significant when *p *< .05.

## 3 RESULTS

### 3.1 Rh2 treatment exerts antidepressant‐like feasibility in the behavioral experiments

**FIGURE 1:**
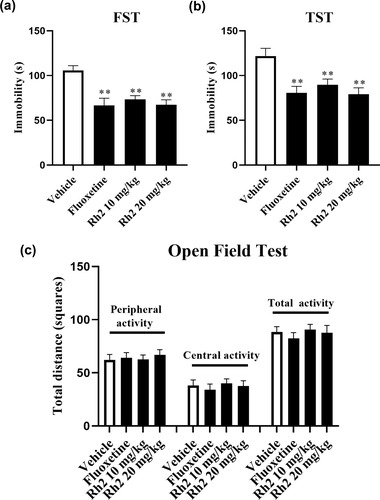
Rh2 improved depression‐like behavior without affecting the locomotor activity of mice. The forced swim test (FST) (a) and the tail suspension test (TST) (b) showed that the immobility time of mice taken by the Rh2 and fluoxetine groups was lower than the vehicle. The open field test (OFT) (c) showed that neither Rh2 nor fluoxetine groups affected the locomotor activity of mice. Data are shown as mean ± SEM. ^**^ *p *< .01 when compared to the vehicle, *n* = 10. The Comparisons were made by one‐way analysis of variance (ANOVA) followed by Tukey's test

Behavioral tests involving the FST and TST were used to evaluate the antidepressant activity in mice (Cryan et al., 2005; Yankelevitch‐Yahav et al., 2015). Compared with the untreated vehicle group, the FST and TST results demonstrated that the groups treated with fluoxetine and Rh2 significantly reduced the immobility time of the mice (Figure 1a,b). FST data revealed a significant effect of Rh2 treatment (ANOVA: *F*_3, 36_ = 25.428, *p *< .01) similar to the TST (ANOVA: *F*_3, 36_ = 34.516, *p* < .01). To eliminate the effects of enhancing locomotor activity in mice after Rh2 treatment (Guan et al., 2021), the OFT was performed later. Fluoxetine or Rh2 showed no significant differences in all the tested mice, indicating that neither Rh2 nor fluoxetine affected the locomotor hyperactivity of mice (Figure 1c). Therefore, Rh2 displayed antidepressant‐like potential based on the FST and TST.

### 3.2 Effects of Rh2 on depression‐like behaviors in CUMS‐exposed mice

**FIGURE 2:**
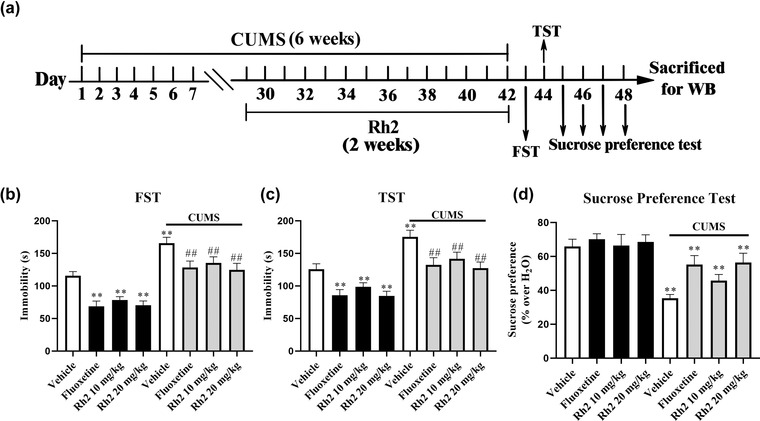
Rh2 treatment remarkably ameliorated the depression‐like behaviors in chronic unpredictable mild stress (CUMS)‐induced mice. The timeline of the experimental design (a). The less immobility duration was taken by the (CUMS + Rh2)‐treated mice than the CUMS‐induced mice in the forced swim test (FST) (b) and in the tail suspension test (TST) (c). The sucrose preference test (d) showed the (CUMS + Rh2)‐treated mice consumed higher sucrose preference than the CUMS‐treated mice. Data are presented as the means ± SEM, *n* = 10. ^**^ *p *< .01 when compared to the Vehicle; ^##^ *p < *.01 when compared to the CUMS. The comparisons were made by two‐way analysis of variance (ANOVA) followed by Bonferroni's test

CUMS is a rodent model of depression and used to simulate the main symptoms of depression in mice (Antoniuk et al., 2019; Willner, 2017). Behavioral evaluations in the FST, TST, and SPT were also performed on days 43 to 48 to demonstrate the antidepressant‐like effects of Rh2 against CUMS. Compared to CUMS mice, Rh2 administration markedly shortened the immobility time in the FST and TST and notably improved sucrose consumption (Figure 2b–d). For the FST data (ANOVA: [interaction: *F*_3, 72_ = 24.783, *p *< .01; CUMS: *F*_1, 72_ = 37.594, *p *< .01; Rh2: *F*_3, 72_ = 18.149, *p *< .01]). For the TST data (ANOVA: [interaction: *F*_3, 72_ = 19.257, *p *< .01; CUMS: *F*_1, 72_ = 30.226, *p *< .01; Rh2: *F*_3, 72_ = 25.163, *p *< .01]). A significant difference in the SPT was found (ANOVA: [interaction: *F*_3, 72_ = 15.249, *p* < .01; CUMS: *F*_1, 72_ = 23.473, *p* < .01; Rh2: *F*_3, 72_ = 14.464, *p* < .01]).

### 3.3 Effects of Rh2 treatment on the activation of the BDNF signaling pathway in the hippocampus of CUMS‐exposed mice

**FIGURE 3:**
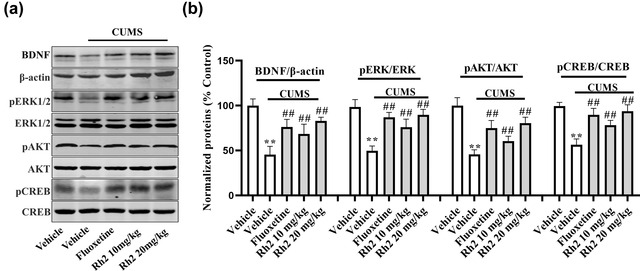
Rh2 promoted BDNF‐CREB signaling cascade in the hippocampus in the model of chronic unpredictable mild stress (CUMS). BDNF, ERK, AKT, and CREB protein levels were assessed by western blotting in the hippocampus. All data are expressed as the means ± SEM, *n* = 5. ^**^ *p *< .01 when compared to the vehicle; ^##^ *p < *.01 when compared to the CUMS. The comparisons were made by two‐way analysis of variance (ANOVA) followed by Bonferroni's test

Subsequently, the levels of the BDNF protein signaling pathways in the hippocampus were determined by western blotting. The relative levels of BDNF, pERK, pAKT, and pCREB proteins were lower in tissue homogenates from the hippocampus in the CUMS group. However, the CUMS‐exposed changes in protein were considerably increased by exposure to Rh2, and fluoxetine also showed the same effect at a dose of 20 mg/kg. BDNF (ANOVA: [*F*_3, 17_ = 29.247, *p* < .01]), ERK (ANOVA: [*F*_3, 17_ = 28.124, *p* < .01]), AKT (ANOVA: [*F*_3, 17_ = 24.173, *p* < .01]), and CREB (ANOVA: [*F*_3, 17_ = 28.443, *p* < .01]) were indicated in Figure 3.

### 3.4 Rh2 administration prevented CUMS‐induced decreases in neurogenesis

**FIGURE 4:**
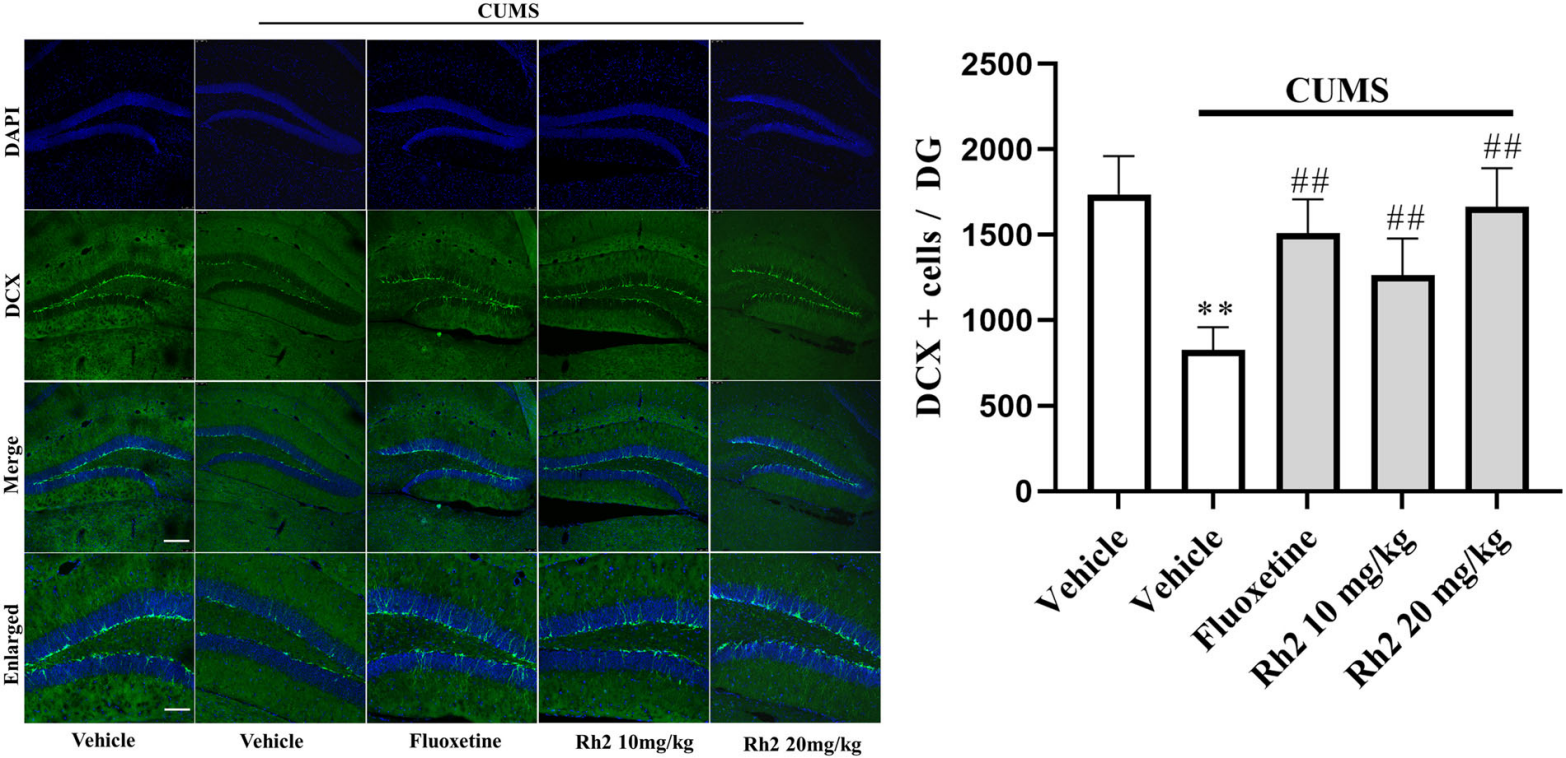
Rh2 treatment revert the hippocampal neurogenesis in the chronic unpredictable mild stress (CUMS) depression model. Immunofluorescences images containing of the DCX in green. Scale bar: 100 μm. All data are shown as the means ± SEM, *n* = 5. ^**^ *p *< .01 when compared to the vehicle; ^##^ *p < *.01 when compared to the CUMS. The comparisons were made by two‐way analysis of variance (ANOVA) followed by Bonferroni's test

Depression is often accompanied by behavior despair and hippocampal neurogenesis reduction, which is effectively reversed by common antidepression drugs, including fluoxetine and citalopram (Boldrini et al., 2012; Dranovsky & Hen, 2006). DCX is a microtubule‐associated protein that is a marker of cellular growth and is expressed in newborn neurons in the DG (Brown et al., 2003). As presented in Figure 4, a lower number of DCX cells were counted in the CUMS group than in the vehicle group in the DG region, while the decreased number of DCX cells was completely reversed by Rh2 treatment (ANOVA: [interaction: *F*_3, 17_ = 29.971, *p *< .01; CUMS: *F*_1, 17_ = 40.276, *p *< .01; Rh2: *F*_3, 17_ = 25.824, *p *< .01]).

### 3.5 Blockade of BDNF‐TrkB signaling abolished the antidepressant effects of Rh2 in CUMS‐exposed mice

**FIGURE 5:**
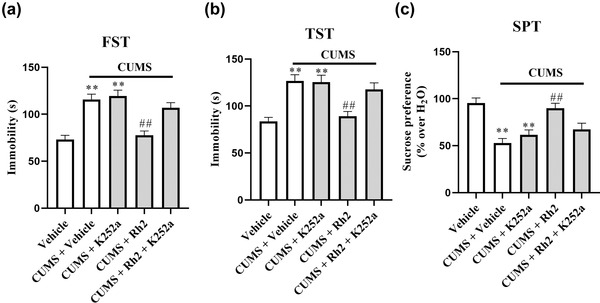
K252a decreased the antidepressant action of Rh2 in chronic unpredictable mild stress (CUMS)‐stressed mice. Co‐treatment with (K252a + Rh2) of the mice against CUMS showed the longer immobility time than the (CUMS + Rh2)‐treated mice in the forced swim test (FST)(a) and tail suspension test (TST) (b). K252a blocked the reversing effects of Rh2 on sucrose consumption of the CUMS‐treated mice (c). All data are expressed as the means ± SEM, *n* = 10. **p < .01 when compared to the Vehicle; ^##^ *p* < .01 when compared to the CUMS. The comparisons were made by two‐way analysis of variance (ANOVA) followed by Bonferroni's test

To explore whether hippocampal BDNF‐TrkB signaling contributes to the antidepressant effects of Rh2, we coinjected CUMS‐treated mice with Rh2 (20 mg/kg), and K252a, a specific inhibitor of the BDNF receptor (TrkB), was used (H. C. Yan et al., 2010). K252a (25 μg/kg) and Rh2 (20 mg/kg, 1 h later) were injected into mice for 2 weeks, and then behavioral tests (FST, TST, and SPT) were conducted. K252a significantly blocked the antidepressant effects of Rh2 in CUMS‐exposed mice in the FST, TST, and SPT (Figure 5a–c).

**FIGURE 6:**
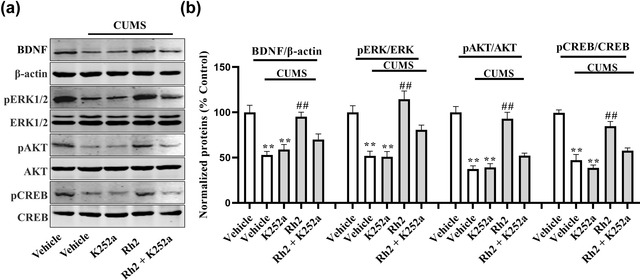
The enhancing influence of Rh2 on the expression of BDNF‐CREB signaling pathway was blocked by the inhibitor K252a in mice exposed to chronic unpredictable mild stress (CUMS). BDNF, pERK/ERK, pAKT/AKT, and pCREB/CREB protein levels were lower in (CUMS + Rh2 + K252a)‐treated mice than the (CUMS + Rh2)‐treated mice. The obtained data are expressed as the means ± SEM, *n* = 5. ^**^ *p *< .01 when compared to vehicle; ^##^ *p < *.01 when compared to CUMS. The comparisons were made by two‐way analysis of variance (ANOVA) followed by Bonferroni's test

**FIGURE 7:**
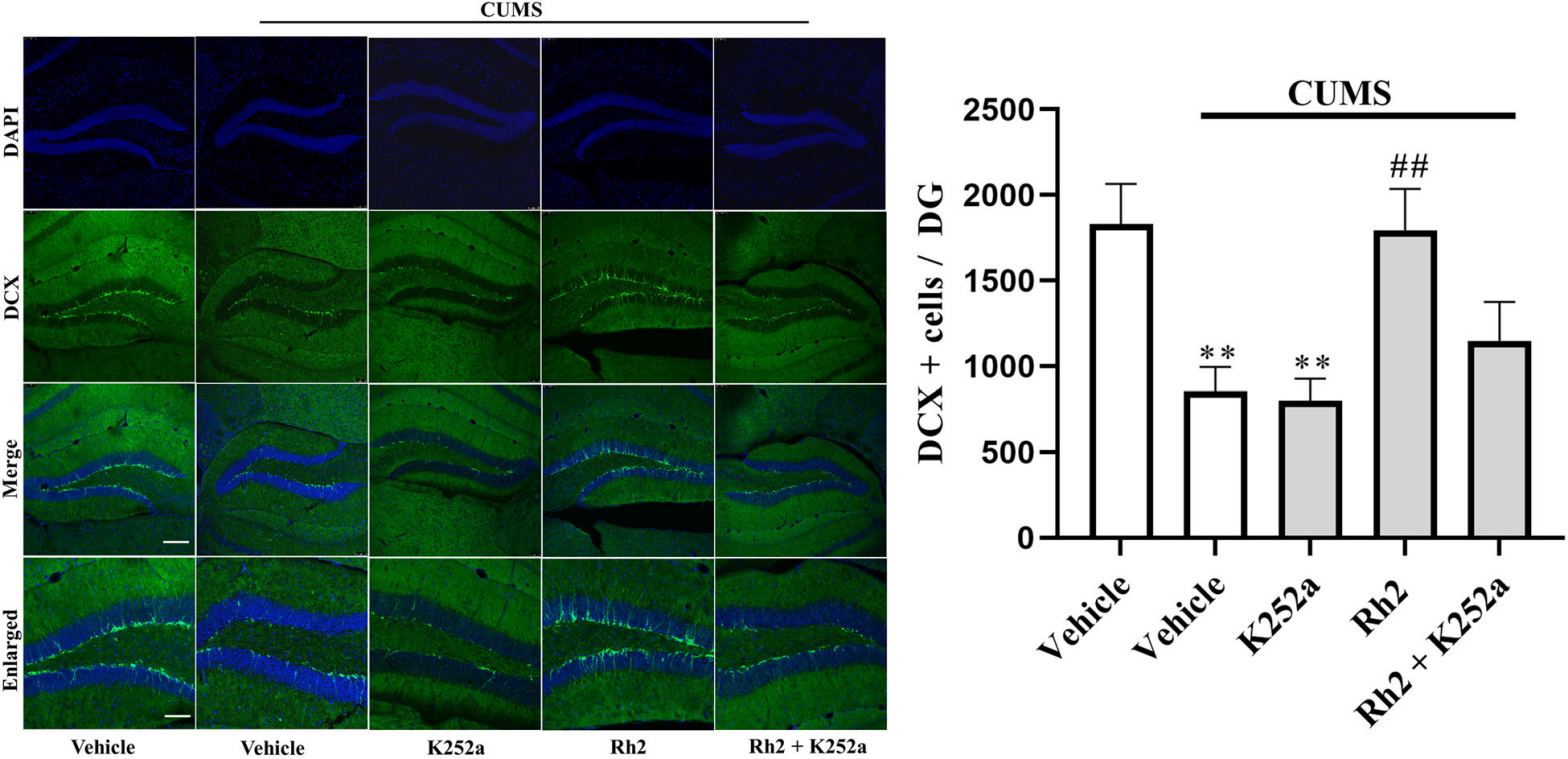
K252a blocked the enhancing effects of Rh2 on neurogenesis. Compared to the (chronic unpredictable mild stress [CUMS] + Rh2)‐treated group, the increase of DCX^+^ neurons in the DG of mice exposed to CUMS were significantly blocked by K252a. Scale bar: 100 μm. The results of analysis are expressed as the means ± SEM, *n* = 5. ^**^ *p *< .01 when compared to vehicle; ^##^ *p < *.01 when compared to CUMS. The comparisons were made by two‐way analysis of variance (ANOVA) followed by Bonferroni's test

Next, the impact of K252a infusion on the BDNF‐TrkB signaling pathway and the immunoreactivity of DCX after Rh2 administration in mice (exposed to CUMS) were determined. It was observed that (Rh2 + K252a) treatment inhibited the effect of Rh2 on the expression of BDNF, p‐ERK, p‐AKT, and p‐CREB in the hippocampus of CUMS‐exposed mice (Figure 6). Similarly, Figure 7 indicates that compared to the Rh2 group, the increased numbers of DCX‐positive cells in the DG were reversed by the (K252a + Rh2) group in the mice (exposed to CUMS). To summarize, the hippocampal BDNF‐CREB system is required for the antidepressant mechanism of Rh2.

## 4 DISCUSSION

In our research, we demonstrated that Rh2 exerted neuroprotective effects in chronic stress mice and has potential in the treatment of depression. We carried out a series of behavioral tests, and the FST and TST are two of the most extensively used tests for evaluating different depressive‐like behaviors (Cryan et al., 2005; Petit‐Demouliere et al., 2005) and screening antidepressants. The SPT is also commonly used to analyze depressive‐like behavior since this test provides information relating to anhedonia, one of the main depressive‐like symptoms (Goñi‐Balentziaga et al., 2018). As a widely applied animal model for depression, the CUMS model presents many core depressive behaviors, including helplessness, anhedonia, and reduced locomotor activity (Z. Zhang et al., 2020). In particular, K252a, a TrkB antagonist was injected into mice exposed to CUMS and completely blocked the BDNF‐TrkB signaling cascade.

Rh2 is a major bioactive constituent of the traditional Chinese medicine ginseng with multiple pharmacological effects, such as antitumor, antifatigue, and antimicrobial effects, along with the ability to enhance immunity (M. Wang et al., 2017; B. Yan et al., 2018; X. Yang et al., 2017). Recent studies have reported that ginsenoside may improve depressive behavior and induce neuroprotective effects in rodents. For example, J. Wang et al. (2016) reported that mice receiving Rh2 treatment showed a significant improvement in their behavioral tests and increased the survival time of CRC‐mice. Chen et al. showed that (24*R*)‐pseudoginsenoside HQ (*R*‐PHQ) significantly reduced the immobility time of mice in the FST and TST and produced promising antidepressant‐like effects in mice. These effects may be related to the Sirt1/NF‐κB or BDNF/TrkB signaling pathways (Chen et al., 2019). H. Zhang et al. (2016) provided evidence that ginsenoside Rb3 exerted antidepressant effects by promoting the monoamine neurotransmitter system. Although the mechanisms by which Rh2 exerts its neuroprotective effects in depression remain largely uncharacterized, our study indicated that as well as the ginsenoside derivatives described above, Rh2 also possesses antidepressant‐like efficacy. Over recent years, some studies have clearly supported a relationship between the neuroprotective effect of Rh2 and its antioxidant activity. Hsieh et al. (2018) suggested that Rh2 ameliorated lipopolysaccharide (LPS)‐induced oxidative stress by regulating signaling pathways (HO‐1/Trx‐1/KAP‐1/Nrf2) in mice. In another study, Rh2 suppressed oxidative stress, had antioxidant activity in vivo, and restored the balance of the antioxidant defense system (Qi et al., 2019). Therefore, these results suggest that Rh2 has potential as a new antidepressant candidate to treat depression in clinical practice.

BDNF is associated with adult neurogenesis and reduced levels of BDNF have been implicated in depression (Szuhany & Otto, 2020). In addition to depression, BDNF also represents a promising therapeutic agent for Parkinson's disease and epilepsy while also providing cardiovascular protection, and response to exercise (T. W. Lin et al., 2020; Palasz et al., 2020; Trombetta et al., 2020). Therefore, Rh2 may exert further pharmacological effects involving BDNF; this possibility needs to be investigated further. By searching the existing literature, we found that mice receiving Rh2 treatment exhibited significantly improved behaviors in the FST, TST, and SPT. These effects appear to be related reductions in depression‐associated cytokines, such as tumor necrosis factor‐alpha (TNF‐α), interleukin‐18 (IL‐18), and IL‐6 (J. Wang et al., 2016). It is important to mention that depression is accompanied not only by BDNF dysfunction and “depression‐like” symptoms but also by proinflammatory cytokines (Clark et al., 2009). Many antidepressants, including notoginsenoside R1, *Hericium erinaceus*, lipopolysaccharide, and baicalin, have been shown to reverse the positive effects on the expression of TNFα, caspase‐3, and IL‐6 in the hippocampus against CUMS‐induced animals (Chong et al., 2019; Guo et al., 2019; B. Zhang, Wang, et al., 2019). In addition, some reports have shown a negative correlation between the expression levels of TNFα, caspase‐3, IL‐6, and BDNF in the brain (Liu et al., 2017; L. Wang, Wei, et al., 2020; Y. Zhang et al., 2018). Collectively, we propose that Rh2 produces antidepressant effects and enhances hippocampal BDNF expression by inhibiting the expression levels of TNFα, caspase‐3, and IL‐6 in mice; this possibility requires further explanation.

The present study has some limitations that need to be considered. First, we used a CUMS model of depression. Apart from CUMS, there are some other established models of depression such as chronic restraint stress and chronic social defeat stress. Furthermore, although depression is accompanied by dysfunction in the BDNF‐TrkB system and neurogenesis, many other pathological symptoms including neuroinflammation, the HPA axis and monoaminergic deficiency are also involved (Blier, 2016; Krishnan & Nestler, 2008). It is possible that Rh2 might ameliorate these symptoms. Furthermore, we observed that BDNF plays an important role in other central nervous system (CNS) disorders in addition to depression, such as schizophrenia and Parkinson's disease (W. Yang et al., 2020). We plan to investigate these possibilities in our future research. Collectively, our data provide more insights into the pharmacological effects of Rh2, which will contribute to the development of the next generation of more effective and safer antidepressants.

## 5 CONCLUSIONS

These findings indicate that Rh2 has antidepressant effects by regulating the BDNF/CREB signaling.

## CONFLICT OF INTEREST

The authors declare no conflict of interest.

## AUTHOR CONTRIBUTIONS

Wei Guan and Wei Zhang designed the research. Wei Guan wrote and amended the manuscript. Chun‐hui Ji, Lin‐sheng Shi, and Yue Liu performed the behavioral experiments, and Jiang‐Hong Gu and Wen‐Qian Tang conducted the molecular biology experiments. Lin‐sheng Shi and Wen‐Qian Tang organized and analyzed the data. All authors read and gave the final approval.

### PEER REVIEW

The peer review history for this article is available at https://publons.com/publon/10.1002/brb3.2705

## Data Availability

Some or all data, models, or code generated or used during the study are available from the corresponding author upon request.
